# Kinetic Modelling of GlmU Reactions – Prioritization of Reaction for Therapeutic Application

**DOI:** 10.1371/journal.pone.0043969

**Published:** 2012-08-27

**Authors:** Vivek K. Singh, Kaveri Das, Kothandaraman Seshadri

**Affiliations:** Innovative Medicine for Infection (iMed Infection), AstraZeneca India Pvt. Ltd., Bangalore, Karnataka, India; Montana State University, United States of America

## Abstract

*Mycobacterium tuberculosis*(*Mtu*), a successful pathogen, has developed resistance against the existing anti-tubercular drugs necessitating discovery of drugs with novel action. Enzymes involved in peptidoglycan biosynthesis are attractive targets for antibacterial drug discovery. The bifunctional enzyme mycobacterial GlmU (Glucosamine 1-phosphate N-acetyltransferase/ N-acetylglucosamine-1-phosphate uridyltransferase) has been a target enzyme for drug discovery. Its C- and N- terminal domains catalyze acetyltransferase (rxn-1) and uridyltransferase (rxn-2) activities respectively and the final product is involved in peptidoglycan synthesis. However, the bifunctional nature of GlmU poses difficulty in deciding which function to be intervened for therapeutic advantage. Genetic analysis showed this as an essential gene but it is still unclear whether any one or both of the activities are critical for cell survival. Often enzymatic activity with suitable high-throughput assay is chosen for random screening, which may not be the appropriate biological function inhibited for maximal effect. Prediction of rate-limiting function by dynamic network analysis of reactions could be an option to identify the appropriate function. With a view to provide insights into biochemical assays with appropriate activity for inhibitor screening, kinetic modelling studies on GlmU were undertaken. Kinetic model of *Mtu* GlmU-catalyzed reactions was built based on the available kinetic data on *Mtu* and deduction from *Escherichia coli* data. Several model variants were constructed including coupled/decoupled, varying metabolite concentrations and presence/absence of product inhibitions. This study demonstrates that in coupled model at low metabolite concentrations, inhibition of either of the GlmU reactions cause significant decrement in the overall GlmU rate. However at higher metabolite concentrations, rxn-2 showed higher decrement. Moreover, with available intracellular concentration of the metabolites and *in vivo* variant of model, uncompetitive inhibition of rxn-2 caused highest decrement. Thus, at physiologically relevant metabolite concentrations, targeting uridyltranferase activity of *Mtu* GlmU would be a better choice for therapeutic intervention.

## Introduction

Tuberculosis (TB) is an infectious disease caused by *Mycobacterium tuberculosis* (*Mtu*) and has plagued humans for centuries. In 2009 alone, 1.7 million people died from TB and 9.4 million new TB cases were reported [1]. The burden from TB is further compounded by the emergence of multidrug-resistant (MDR) and extensively drug-resistant (XDR) strains of *Mtu* that are resistant to first line and first and second line anti-TB drugs respectively. Consequently, there is a pressing need for novel anti-TB drugs that can inhibit novel targets such that MDR and XDR strains can be tackled along with the drug sensitive *Mtu* strains.

**Figure 1:**
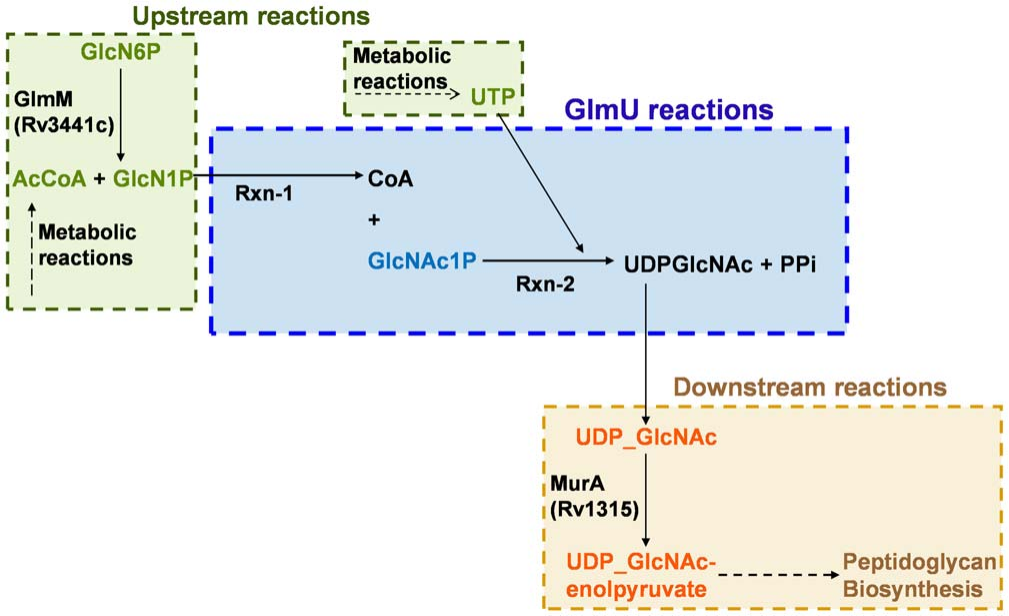
Pathway context of GlmU-catalyzed reactions.



The mycobacterial cell wall consists of covalently linked complex of mycolic acids and arabinogalactan, which is linked to peptidoglycan. The pathway for the peptidoglycan biosynthesis has been the target for several antibacterial agents such as cycloserine and fosfomycin [2], [3]. In this pathway, UDP-N-acetyl-D-glucosamine (UDPGlcNAc) is an essential precursor for peptidoglycan and is synthesized by the enzyme - glucosamine-1-phosphate-acetyltransferase/N-acetylglucosamine-1-phosphate-uridyltransferase (GlmU) (Figure 1). GlmU exists as a bifunctional enzyme in many bacteria including *Mtu*, catalysing two consecutive reactions – first, acetyltransferase reaction converting alpha-D-glucosamine-1-phosphate (GlcN1P) to N-acetyl-alpha-D-glucosamine-1-phosphate (GlcNAc1P) coupled with the conversion of acetyl coenzyme A (AcCoA) to coenzyme A (CoA) and the second, uridyltransferase reaction converting GlcNAc1P to UDPGlcNAc utilizing UTP as uridyl group donor and releasing pyrophosphate (PPi) as a product (see scheme 1) [4], [5]. The acetyltransferase (rxn-1; EC 2.3.1.157) and uridyltransferase (rxn-2; EC 2.7.7.23) activities of this enzyme are catalyzed by its C- and N-terminal domains respectively [4]. This bifunctional nature of GlmU offers an increased opportunity for inhibition, given the fact that the enzyme has already been suggested as an attractive target for TB drugs [6], [7]. However, since the inhibition of these reactions may not lead to the same level of overall decrement in the GlmU rate, it is important to determine the reaction with maximal impact so as to design assays appropriately to target this enzyme.

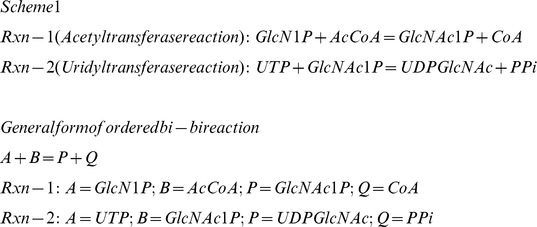



Kinetic modelling offers a computational means to assess the dynamic behaviour of biochemical reaction systems and has been employed in the past to select drug targets by simulating the effect of enzyme inhibition on the dynamics of reaction systems [8], [9], [10]. It is interesting to note that recently, although genome-scale kinetic models have also been attempted [11], [12], typically kinetic models are restricted to a small set of reactions [13], [14] due to their requirement for enzyme kinetic parameters. GlmU-catalysed reactions forms an ideal system for such kinetic modelling based analysis, so as to evaluate their relative control on the overall GlmU rate and to identify the one whose inhibition would cause maximal effect on the overall rate.

Reported in this work is the construction of a kinetic model of GlmU-catalyzed reactions in *Mtu* and its application to GlmU reactions for achieving therapeutic goal. Furthermore, on the basis of modelling studies, the preferred mode of inhibition and the initial metabolite concentrations for the design of an *in vitro* assay were also proposed, which can bias the assay towards the selection of specific type of inhibitor against the reaction of choice.

## Methods

### Biochemical reactions in the model

The reactions catalysed by GlmU i.e. acetyltransferase (rxn-1) and uridyltransferase (rxn-2) together constitute the set of biochemical reactions focussed on in the present model (see scheme 1). The stoichiometric equations for the reactions were obtained from KEGG [15], [16], [17] and cross-verified from literature sources (2), [4]. As can be noticed from scheme 1, the functioning of rxn-2 is dependent on that of rxn-1 by virtue of sharing of intermediate GlcNAc1P between the reactions, which is a product of rxn-1 and acts as a substrate for rxn-2. It is noteworthy that GlcNAc1P is released from the acetyltransferase domain prior to binding to the uridyltransferase domain of GlmU [18], thus, eliminating the possibility of substrate (GlcNAc1P) channelling.

### Rate equations

It is evident that both the GlmU reactions (scheme 1) follow Michaelis Menten ordered bi-bi mechanism [19], [4] involving an obligatory order of binding of substrates to the enzyme. In GlmU rxn-1, GlcN1P is the first substrate to bind to the free enzyme (E) followed by the binding of AcCoA to E-GlcN1P complex. A similar binding order was presumed in the reverse direction wherein GlcNAc1P binds to the free enzyme followed by the binding of CoA. In GlmU rxn-2, UTP binds to the free enzyme followed by the binding of GlcNAc1P and in the reverse direction, UDPGlcNAc binds to the free enzyme followed by the binding of PPi [4]. The binding of products (substrates for reverse reactions) to the enzyme was taken into account so as to include the effect of product inhibition in the model.

**Figure 2:**
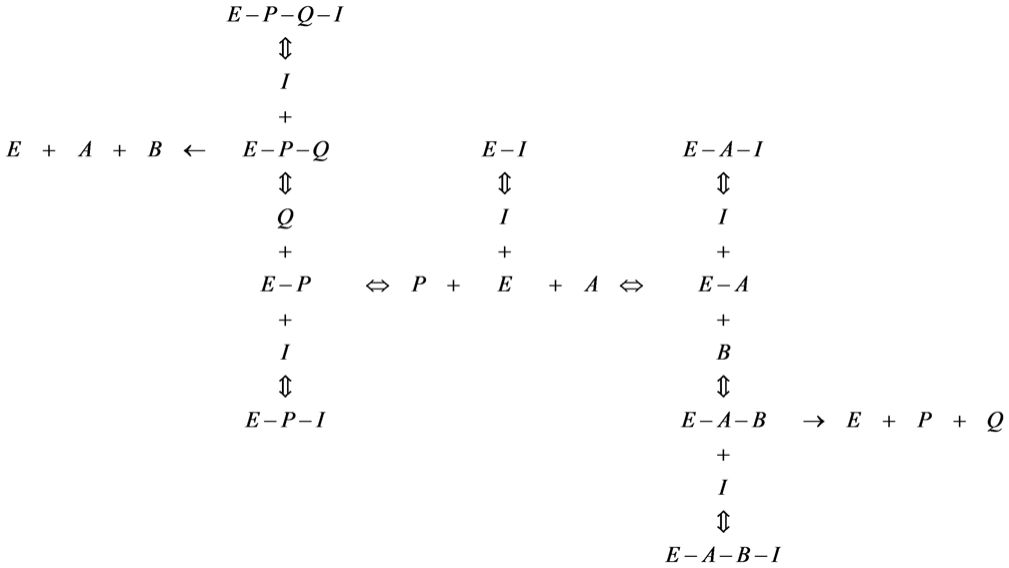
Equilibria between enzyme species for ordered bi-bi mechanism of enzymatic reaction. *A, B*  = First and second substrate of enzyme *E*; *P, Q*  = First and second substrates for the reverse reaction, their binding to enzyme accounts for product inhibition; *I*  = Different types of hypothetical inhibitor, whose type is determined by the form of enzyme it binds to: *I* binding to free *E* (forming *E-I* complex) is a competitive inhibitor with respect to *A*, *I* binding to *E-A* complex (forming *E-A-I* complex) is uncompetitive inhibitor with respect to *A* and *I* binding to *E-A-B* complex (forming *E-A-B-I* complex) is uncompetitive inhibitor with respect to both *A* and *B*; *K_ic_*  = Inhibition constant of hypothetical competitive inhibitor; *K_iu_<metabolite>_*  = Inhibition constant of hypothetical uncompetitive inhibitor where the inhibitor behaves uncompetitive against the metabolite indicated within <>.

Assigning the substrates and products of GlmU reactions to the general form of ordered bi-bi reaction as depicted in scheme 1, a general rate equation was derived based on rapid equilibrium kinetics following the method outlined by Segel [20]. The reaction equilibria constructed (see Figure 2) to derive the rate equation included: (1) the binding of products to the enzyme and (2) the binding of different types of hypothetical inhibitors (I) to the enzyme, such that the derived general rate equation can account for reverse reaction, product inhibition and inhibition by various types of hypothetical inhibitors depending on the values assigned to the terms contained in the rate equation.

Briefly, the steps for the derivation of a rate equation involve:

(1) Writing a general rate equation based on the concentration of enzyme complexes that yield products
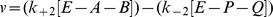
where *k_+2_* and *k_−2_* are turnover numbers for forward and reverse reactions respectively.

(2) Dividing both sides of this equation by the total enzyme concentration, **[** ***E_t_*** **]**, which on the right-hand side of the equation is expressed as the sum of the concentration of all enzyme species:
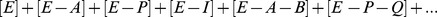



(3) Expressing the concentrations of all the enzyme species in terms of free enzyme concentration derived on the basis of equilibria (see Figure 2):
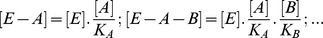
where *K_<metabolite>_*  = Michaelis constant of the metabolite specified between <>

(4) Performing algebraic operations, moving [*E_t_*] to the right-hand side and defining 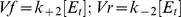 the equation becomes:



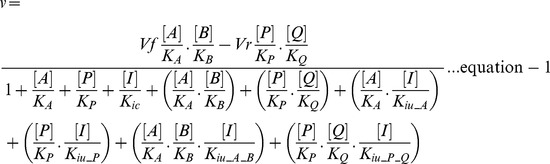



In the rate equation (equation-1), the metabolite and inhibitor notations such as [*A*], [*B*], [*P*], [*Q*] and [*I*] represent the concentration of respective metabolites and inhibitor; *Vf*  = maximal rate in forward direction; *Vr*  = maximal rate in reverse direction; *K_ic_*  = inhibition constant of hypothetical competitive inhibitor; *K_iu_<metabolite>_*  = inhibition constant of a hypothetical inhibitor which is uncompetitve against the metabolite indicated within <>. Further, the terms representing the ratios of inhibitor concentration to inhibition constant in the rate equation were substituted by variables as follows: *a_glmu_rxn  = *[*I*]*/K_ic_*; *b_A_glmu_rxn  = *[*I*]*/K_iu_A_*; *b_P_glmu_rxn  = *[*I*]*/K_iu_P_*; *b_A_B_glmu_rxn  = *[*I*]*/K_iu_A_B_*; and *b_P_Q_glmu_rxn  = *[*I*]*/K_iu_P_Q_*. This representation eliminates the need for any assumption on inhibitor concentration or inhibition constants and also provides a normalised measure of inhibition strengths of various types of hypothetical inhibitors. It can be noticed that when values of all such variables are zero, the rate equation represents an uninhibited enzymatic reaction, while the effect of inhibition in the model can be simulated by assigning non-zero values to these variables. Equation-2 is the resulting general rate equation after this manipulation. The rate equations of GlmU reactions were deduced from this general rate equation by substituting the corresponding metabolites for *A, B, P* and *Q* (as provided in scheme 1) and were used for the model.



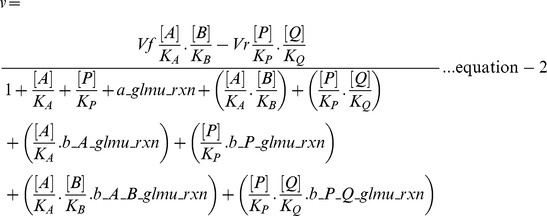



### Kinetic parameters of the model

The dynamics of GlmU-catalyzed reactions can be simulated by solving the rate equations; however, to solve them, values of all the terms on the right-hand side of the equations (such as *Vf*, *Vr*, *K_M_*) need to be assigned. Kinetic parameters constitute a part of all the terms on right-hand side of rate equations and are described in this section. The terms dealing with initial concentration of metabolites are discussed in later section.

The kinetic parameters for rxn-2 were available for *Mtu* from UTP and GlcNAc1P *in vitro* concentration response experiments carried out in AstraZeneca (unpublished). However, substituting these values in the rate equations of the model did not quantitatively reproduce the experimentally obtained *in vitro* concentration response curves of UTP and GlcNAc1P. The non-agreement is due to the difference in the rate equations used in the experiment and in the model. While the experimentally obtained data is fitted to simple Michaelis-Menten (MM) equation, the model was simulated with ordered bi-bi mechanism in accordance to previous reports ([19], [4]). To overcome this issue, the parameter values that could reproduce the experimental GlcNAc1P concentration response curve were used for all further simulations (see Table 1 for the parameters used for simulation and Table S1 for information on the derivation of kinetic parameters). GlcNAc1P concentration response curve was chosen for alignment of parameter values because the other substrate for GlmU rxn-2, UTP, is present in the intracellular milieu at high concentration ( = 8.3mM (see Table 2)). At this concentration, UTP would be available to the enzyme at saturating level, which is also the case while performing GlcNAc1P concentration response experiment. Further, to obtain the best fit with the GlcNAc1P concentration curve, parameter estimation functionality of COPASI [21] was used. The input data to it were: (1) the list of GlcNAc1P concentrations and their corresponding reaction rates obtained experimentally; (2) the parameter values obtained experimentally and the range in which they can be varied to minimize the error between the experimental and simulated reaction rates. Following were the range of parameter values used: 0.015nmol/min ≤ *Vf* ≤0.020nmol/min; 0.04mM ≤ *K_UTP_* ≤0.04mM; 0.01mM ≤ *K_GlcNAc1P_* ≤0.1mM and the experimentally obtained parameter values are: *Vf*  = 0.020nmol/min; *K_UTP_*  = 0.04mM; *K_GlcNAc1P_*  = 0.04mM. Evolutionary programming was used as the method of optimization with number of generations  = 200, population size  = 20 and random number generator  = 1 [21]. The best fit (root mean square error  = 0.51) was obtained with the parameter values (*Vf*  = 0.020nmol/min; *K_UTP_*  = 0.04mM; and *K_GlcNAc1P_*  = 0.033mM). These values were used for further simulations.

**Table 1:** Kinetic parameters used for simulating the model.

Rxn-1 Parameters		Rxn-2 Parameters	
*Vf*	= 0.01089mM/min	*Vf*	= 0.0004mM/min
*K_GlcN1P_*	= 0.061mM	*K_UTP_*	= 0.040Mm
*K_AcCoA_*	= 0.224mM	*K_GlcNAc1P_* a	= 0.033mM
		*K_GlcNAc1P_2_^b^*	= 0.033mM
*Vr*	= 0mM/min	*Vr*	= 0mM/min
*K_GlcNAc1P_*	= 0.003mM	*K_UDPGlcNAc_*	= 0.132mM/min
*K_CoA_*	= 10^9^mM	*K_PPi_*	= 10^9^mM

a This parameter present in coupled model but absent in decoupled model;^b^This parameter present in decoupled model but absent in coupled model; See Table S1 for details on derivation of kinetic parameters.

**Table 2:** Initial metabolite concentrations and the boundary conditions for various variants of model.

Metabolite	Low (mM)	Medium (mM)	High (mM)	Intracellular (mM)	Intracellular conc/*K_M_*	BoundaryCondition^a^	
						*In vivo* model	*In vitro* model
GlcN1P	0.0061	0.061	0.61	0.08	1.31	True	False
AcCoA	0.0224	0.224	2.24	1.71	7.63	True	False
GlcNAc1P	0.0	0.0	0.0	0.082 *^in vivo^;* 0.0 *^in vitro^*	2.48 *^in vivo^;* 0.0 *^in vitro^*	False	False
GlcNAc1P_2^b^	0.0033	0.033	0.33	0.082	2.48	True	False
CoA	0.0	0.0	0.0	1.2 *^in vivo^;* 0.0 *^in vitro^*	-	True	False
UTP	0.004	0.04	0.4	8.3	207.50	True	False
UDPGlcNAc	0.0	0.0	0.0	9.2 *^in vivo^;* 0.0 *^in vitro^*	69.70 *^in vivo^;* 0.0 *^in vitro^*	True	False
PPi	0.0	0.0	0.0	0.5 *^in vivo^;* 0.0 *^in vitro^*	-	True	False

a Boundary condition “True” for a metabolite indicates that its concentration is not determined by the set of reactions even when that metabolite occurs as a substrate or product i.e., the metabolite is on the boundary of the reaction system but is a component of the rest of the model (see [28] for more details); ^b^ This metabolite only present in decoupled version of model; *^in vivo^* Represents *in vivo* variant of model; *^in vitro^* Represents *in vitro* variant of model.

The *K_M_* values for rxn-1 were taken from literature [5], and the *Vf* were abstracted from the experimental data on rxn-2 (as obtained in AstraZeneca (unpublished)) and scaling factor from literature [5] (see Table 1 for the parameters used for simulation and Table S1 for information on the derivation of kinetic parameters).

### Variants of the model

Several model variants were constructed to explore the following possibilities: Presence/absence of product inhibition; Coupled/decoupled model – reaction coupling due to product of rxn-1 acting as a substrate for rxn-2; Low/medium/high/intracellular metabolite concentrations – concentrations of the metabolites kept at *0.1xK_M_*, *K_M_*, *10xK_M_* or intracellular levels; and *In vitro* and *in vivo* model – representing condition in a biochemical assay *vs.* condition inside a cell respectively.

The effect of product inhibition is simulated by assigning the literature derived values [22] of Michaelis constants to relevant products of GlmU reactions in the model, while the absence of product inhibition is simulated by making these Michaelis constants equal to a large number ( = 10^9^mM) such that the affinity of the enzyme for the products reduces to negligible. Product inhibition of rxn-1 by GlcNAc1P and rxn-2 by UDPGlcNAc was accounted for in the model [22] (see Table S1 for information on the derivation of kinetic parameters).

The model construction described here behaves as a coupled model because the intermediate GlcNAc1P acts as a product of rxn-1 and a substrate for rxn-2. Coupled model was constructed so as to represent the dependence of rxn-2 on rxn-1, due to a product of rxn-1, GlcNAc1P, serving as a substrate for rxn-2. The evidence for coupling comes from the fact that *Mtu* does not have any other route for synthesizing GlcNAc1P except GlmU rxn-1 (KEGG [15], [16], [17]). On the other hand, decoupled model was constructed with the aim of studying the behaviour of each GlmU reaction independent of the other GlmU reaction, which is usually done under *in vitro* assays. To construct a decoupled model, one hypothetical metabolite GlcNAc1P_2 was defined. In the decoupled model, this metabolite was made to act as a substrate for rxn-2 while the original metabolite representing GlcNAc1P (formed as a product of rxn-1) cannot serve as a substrate for rxn-2. Thus, in the decoupled model, two variables exist to represent GlcNAc1P: GlcNAc1P and GlcNAc1P_2. The concentrations of these two variables are independent of each other, making the GlmU reactions independent (decoupled) of each other. Concomitant change in rate equation of rxn-2, i.e. GlcNAc1P_2 assigned as second substrate, was made to represent this decoupling.

The concentrations of the metabolites were modulated depending upon the condition the models represented. The concentrations of the precursors and products in the model were kept variable as in a *in vitro* biochemical assay condition or constant as can be expected inside a *in vivo* cellular environment where the precursor substrates would be synthesised by the reactions upstream of GlmU-catalysed reactions and similarly the products would be consumed by the reactions downstream of the GlmU-catalysed reactions (see Figure 1).

### Initial concentrations of the metabolites

Initial concentrations of the metabolites acting only as substrates were kept low (* = 0.1xK_M_*), medium (* = K_M_*), high (* = 10xK_M_*) or equal to intracellular levels (obtained from diverse bacterial sources including *Mycobacterium tuberculosis* ATCC 25618 and *Eco* [23], [24], [25], [26], [27]) to simulate the dynamics of the reaction system under diverse scenarios (see Table 2). Initial concentrations of the metabolites acting only as products (such as CoA, UDPGlcNAc and PPi) were kept equal to zero for the conditions of low, medium or high metabolite concentrations. Under the intracellular metabolite concentration scenario, the concentrations of metabolites were maintained at different levels for *in vitro* and *in vivo* variants of models. For the *in vitro* variant, the concentrations of intermediate metabolites (such as GlcNAc1P) or metabolites that act only as products (such as CoA, UDPGlcNAc and PPi) were initialized to zero (as can be expected under *in vitro* assay condition), while for the *in vivo* variant, the concentrations of such metabolites were initialized to their respective intracellular levels. Furthermore, for the decoupled variant of model, the concentration of GlcNAc1P_2 was kept equal to its intracellular level under both *in vitro* as well as *in vivo* scenario because it acts as a substrate for rxn-2; the concentration of other variable representing GlcNAc1P is maintained as described above. As discussed in the previous section, concentrations of precursors and products were kept constant to represent *in vivo* situation. This was achieved by defining such metabolites as boundary metabolites (i.e. *boundaryCondition ="True”*), which implies that the said metabolites are at the boundary of the reaction system being modelled and their concentrateons are not determined by rate equations even when they participate in reaction(s) (see [28] for more details).

### Simulation of the model

All the above described variants of model are represented in Systems Biology Markup Language (SBML) [28], which is the standard format to represent such Systems Biology models. To perform the simulations, a *Matlab* script was written that reads the SBML file representing the model, performs time course and steady state simulations of the uninhibited model, followed by the time course and steady state simulations of the model with the influence of different types of inhibitors. The simulations were performed with inhibition strength (i.e. *I/Ki* ratio) kept equal to 20, which is a typical ratio of inhibitor concentration and its corresponding inhibition constant. The computations were performed in *Matlab* using the *Matlab* toolboxes – SBML toolbox [29] and SBTOOLBOX2 [30].

## Results and Discussion

### Reproduction of *in vitro* concentration response curve

**Figure 3:**
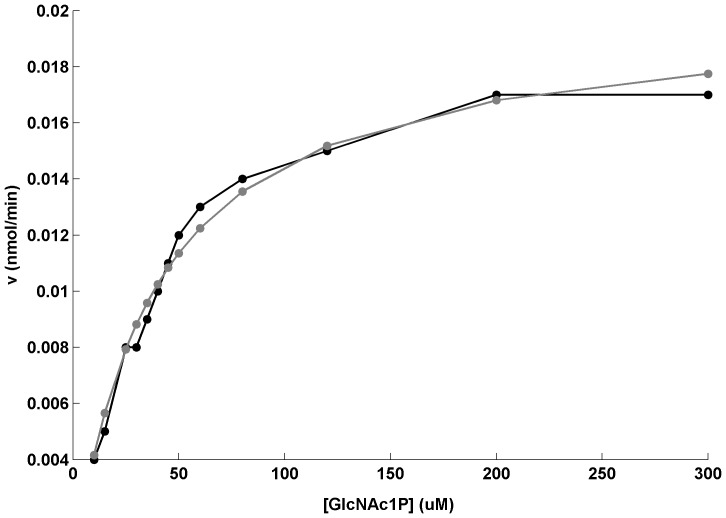
Experimental *vs.* simulated concentration response curves. GlcNAc1P concentration response curve; Curves obtained from experiment: Black; Curves obtained from simulation: Gray; *v*  = Velocity of GlmU rxn-2. Assays were carried out at 25°C in assay buffer containing 50 mM Hepes KOH pH 7.5, 5 mM MgCl2. 5 mM DTT, 0.3 units/ml pyrophosphatase and the phosphate formed was detected using malachite green reagent from Innova Biosciences. For GlcNAc1P KM determination UTP was fixed at 250 µM.

The model aligned to GlcNAc1P concentration response curve was able to reproduce the experimentally observed curve (see Figure 3). The *in vitro* biochemical assay simulation conditions viz., (1) constant concentration of 0.25mM for UTP and GlcNAc1P; (2) no depletion of substrates; and (3) no accumulation of products, were maintained for the simulation.

### Product inhibition leads to quantitative differences in the model dynamics

The influence of product inhibition on the dynamical behaviour of the model was investigated by building separate versions of the model (see “Variants of the model” section of “Methods” for details). The product inhibition on rxn-1 increased progressively with increasing metabolite concentration due to increase in normalized product concentration (*[product]/K_i_product_*) relative to normalised substrate concentration (*[substrate]/K_M_substrate_*) (data not shown). On the contrary, product inhibition on rxn-2 decreased progressively with increasing metabolite concentration due to decrease in normalized product concentration (*[product]/K_i_product_*) relative to normalised substrate concentration (*[substrate]/K_M_substrate_*) (data not shown). As expected, rates of the reactions were higher in the version without product inhibition as compared to the version with product inhibition. Furthermore, the difference caused in the rate of rxn-1 was comparatively higher than that caused in the rate of rxn-2 because the product of former reaction (GlcNAc1P) causes stronger product inhibition on rxn-1 (*K_GlcNAc1P_*  = 0.003mM) compared to that caused by product of latter reaction (UDPGlcNAc) on rxn-2 (*K_UDPGlcNAc_*  = 0.132mM). These phenomena were observed with both, *in vitro* and *in vivo* variants of model. The product inhibition was included for all further simulations owing to experimental evidence [22] and to provide a biological relevance to the simulations.

### Coupled *vs.* decoupled models

Coupled model was constructed to represent the dependence of rxn-2 on rxn-1, representing condition closer to cellular environment. In contrast, decoupled model was constructed to study the behaviour of each GlmU reaction independently, which is usually done under *in vitro* assays. Discussed below is the contrast between the dynamic behaviour of these versions of model in the presence of product inhibition over a wide range of metabolite concentrations.

**Figure 4:**
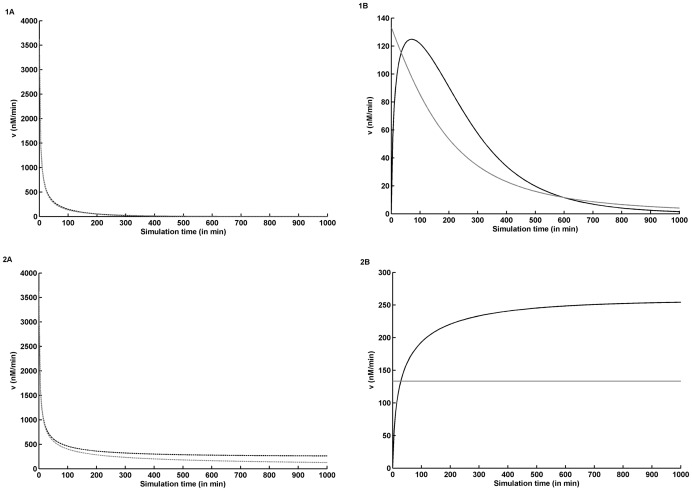
Dynamic behaviour of the rates of GlmU reactions in coupled *vs.* decoupled models. Plots corresponding to medium ( = *K_M_*) metabolite concentrations; *v*  = Rates of GlmU rxn-1 (broken lines) and rxn-2 (solid lines); Panel 1A: *In vitro* variant GlmU rxn-1; Panel 1B: *In vitro* variant GlmU rxn-2; Panel 2A: *In vivo* variant GlmU rxn-1; Panel 2B: *In vivo* variant GlmU rxn-2; Coupled model: Black lines; Decoupled model: Gray lines.

As shown in Figure 4 (panels 1A and 1B), under *in vitro* condition, a major difference observed is that the functioning of rxn-2 begins after a delay in coupled model, which is the time period required for synthesis of GlcNAc1P; in contrast in the decoupled model, rxn-2 begins instantaneously due to already available non-zero concentration of GlcNAc1P_2. This delay shortens with an increase in initial metabolite concentrations. Furthermore, at medium metabolite concentrations, the rate of rxn-2 initially increases with an increase in the GlcNAc1P concentration but eventually drops down to zero due to exhaustion of UTP (another substrate of rxn-2). Such a sharp decrease in the rate of rxn-2 is not observed in decoupled model owing to equal concentration of both its substrates due to their equal *K_M_* in *Mtu*. At high metabolite concentration, sharp decrease in the rate of rxn-2 is not observed in coupled model while decoupled model showed a gradual decrease in rxn-2 rate (data not shown), which can be explained by the drop in GlcNAc1P_2 concentration.

In contrast to *in vitro* condition, *in vivo* simulation showed that the reaction system proceeds towards a steady state in both coupled as well as decoupled models (see panels 2A and 2B of Figure 4). Flux through both reactions in coupled model become equal asymptotically at steady state, while the rates of the reactions in decoupled model remain unequal at steady state. No abrupt drop in rates of any of the reactions is observed as was observed *in vitro* condition. This can be attributed to the apparent constant supply of precursors and constant consumption of products caused by representing the concentrations of precursors and products as constant. Furthermore, rate of rxn-2 in decoupled model remained constant throughout the simulation owing to constant supply and consumption of its reactants and products respectively.

### *In silico* inhibition of GlmU reactions

*In silico* inhibition of each of the GlmU reactions was performed with a goal to prioritize a GlmU reaction, inhibiting which, would cause maximal decrement in the overall GlmU rate. Moreover, the objective of inhibiting GlmU reaction(s) being the disruption of the synthesis of its end-product (i.e. UDPGlcNAc), the rate of rxn-2 was used as a marker for the overall GlmU reaction rate. It must be pointed out that the choice of rxn-2 as a marker for overall GlmU rate was based on the fact that the synthesis of end-product of GlmU reactions would be proportional to the rate of rxn-2.

**Figure 5:**
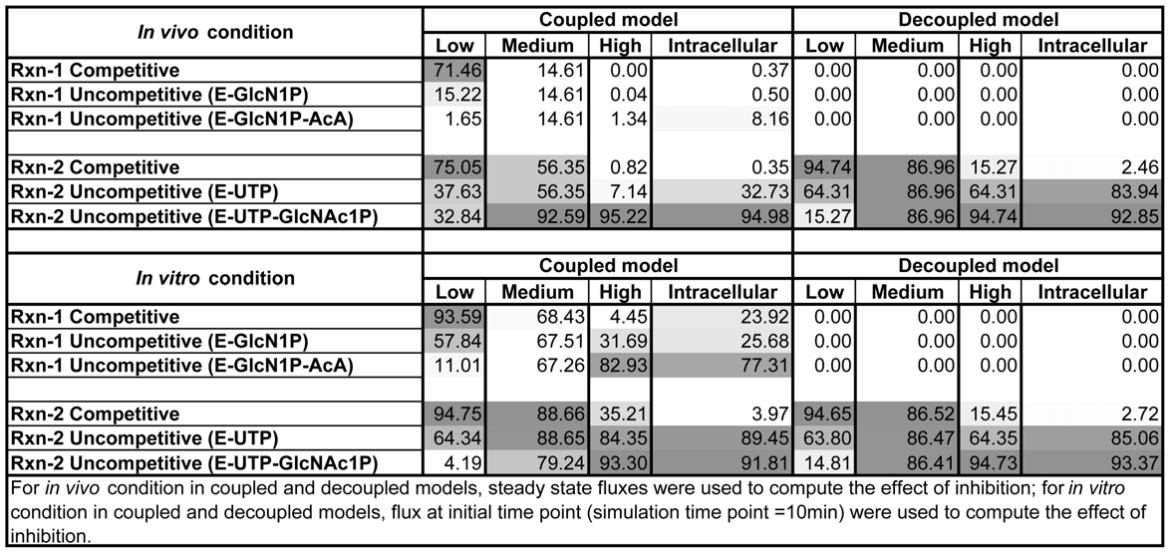
Effect of *in silico* inhibition of GlmU reactions under various conditions. Metabolite concentrations used for simulation: Low (* = 0.1xK_M_*), Medium (* = K_M_*), High (* = 10xK_M_*) and Intracellular levels; Inhibition strength (*I/K_i_* ratio) maintained at 20; Numbers in the figure indicate percent decrement in GlmU overall rate due to various types of inhibition; Linear color-coded scale from Gray to White indicating decreasing level of effect of inhibition on GlmU rate).

In the decoupled model, inhibition of GlmU rxn-1 caused no decrement in the overall Glmu rate. This is due to the fact that functioning of rxn-2 is independent of rxn-1 in the decoupled model. Inhibition of rxn-2 followed expected kinetic behaviour wherein potency of competitive inhibitor decreased with increasing metabolite concentrations and *vice versa* for uncompetitive inhibitors (see top right (for *in vivo* condition) and bottom right (for *in vitro* condition) sections of Figure 5).

Inhibition of GlmU reactions in the coupled model under in vivo condition provided following insights:

#### Inhibitor of either of the GlmU reactions is potent at low ( = 0.1xK_M_) metabolite concentration

As expected, competitive inhibitors against either of the GlmU reactions appeared more potent than uncompetitive inhibitors under low metabolite concentrations. The percent inhibition caused by competitive inhibitors of GlmU reactions is similar (see top left section column “Low” of Figure 5). This indicates that both the reactions have similar control over GlmU rate at low metabolite concentrations, which is also apparent from the computed flux control coefficients (see Table S2) (see [31] for theory of flux control coefficient computation). Flux control coefficient computation also indicated that the control over GlmU rate shifts towards rxn-2 with an increase in metabolite concentrations, which is evident from high potency of inhibitors of rxn-2 at medium and high metabolite concentrations (see below).

#### GlmU rxn-2 inhibition is potent at medium ( = K_M_) and high ( = 10xK_M_) metabolite concentration

With an increase in the initial metabolite concentrations, the relative potency of inhibitors of rxn-2 increased with respect to that of rxn-1 inhibitors. At medium metabolite concentrations, the decrement caused by uncompetitive (against E-UTP-GlcNAc1P complex) inhibition of rxn-2 was the highest, followed by the competitive and uncompetitive (against E-UTP complex) inhibitions of rxn-2, which were equal. The decrement by latter were higher than that caused by competitive and uncompetitive inhibitions of rxn-1, which were equal to each other (see top left section column “Medium” of Figure 5). At high metabolite concentrations, uncompetitive inhibition (against E-UTP-GlcNAc1P complex) of rxn-2 caused highest decrement in GlmU overall rate. All the other types of inhibition of rxn-2 or any type of inhibition of rxn-1 caused low decrement in the GlmU overall rate (see top left section column “High” of Figure 5).

#### Uncompetitive inhibition (against E-UTP-GlcNAc1P complex) of GlmU rxn-2 is the best choice to achieve therapeutic objective

When *in silico* inhibition of GlmU reactions was performed with metabolite concentrations maintained at their intracellular levels (as obtained from diverse bacterial sources including *Mtu* ATCC 25618 and *Eco* [23], [24], [25], [26], [27]), uncompetitive (against E-UTP-GlcNAc1P complex) inhibition of rxn-2 caused the highest decrement in overall GlmU rate. This was followed by the decrement caused by uncompetitive (against E-UTP complex) inhibition of rxn-2, which was rather low (∼33%) (see top left section column “Intracellular” of Figure 5).

The high potency of uncompetitive inhibition (against E-UTP-GlcNAc1P complex) of rxn-2 can be attributed to: (1) high normalised metabolite concentration (defined as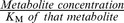) of UTP ( = 207.50) at its intracellular level; and (2) significant normalised metabolite concentration of GlcNAc1P ( = 24.99) during simulation (at steady state).

Furthermore, since the potency of such inhibition is largely dependent on intracellular UTP concentration, which can be expected to be under tight control in cellular environment, the inhibition would not be overcome by compensatory alteration in UTP concentration. Thus, it appears that uncompetitive (E-UTP-GlcNAc1P complex) inhibition of rxn-2 is the best choice to achieve the therapeutic goal of reducing overall GlmU rate to ultimately interrupt the cell wall biosynthesis process in *Mtu*.

The next challenge is to identify uncompetitive inhibitors that can bind to E-UTP-GlcNAc1P complex and inhibit GlmU rxn-2. Easy as it may sound, but finding uncompetitive inhibitors have always been difficult and the present case demands an inhibitor that is required to bind to a pocket other than the two substrate-binding pockets. A recent evidence of the existence of an allosteric binding site in (*Haemophilus influenzae (Hin)*) GlmU uridyltransferase (rxn-2 catalysing) domain [32] offers a cue on the direction in which this study should advance i.e. the allosteric binding site should be exploited using structure-based approaches so as to design the desired uncompetitive inhibitors. In a more recent study performed on *Mtu*, an analogue of GlcN1P was found to be an uncompetitive (against E-GlcN1P complex) inhibitor of GlmU rxn-1 albeit with a poor *Ki* value (18.69mM) [33].

Inhibition of GlmU reactions in coupled model under in vitro condition led to an interesting observation as described below:

#### Inhibited rate greater than uninhibited rate

In general, the inhibition pattern displayed by *in vitro* version of model was similar to *in vivo* model, except for a phenomenon wherein at certain metabolite concentrations, GlmU overall rate in the presence of inhibitor exceeded compared to the absence of inhibitor. This resulted due to slow exhaustion of UTP in inhibited system compared to uninhibited system due to a sheer low rate of reaction in inhibited system. At the initial phase, inhibited rate of rxn-2 is slower than uninhibited (thus conserving UTP), which then pays off after a time point where uninhibited rate of rxn-2 reaches zero while inhibited system continues with the residual UTP. Such phenomenon would usually not be observed *in vitro* assays because the reactions are never allowed to reach such late stage kinetics. This would also not be observed *in vivo* situations because UTP would be supplied for GlmU rxn-2 from UTP synthesizing pathways.

### Prediction of initial metabolite concentrations suitable for *in vitro* biochemical assay for the screening of GlmU rxn-2 inhibitors

As is apparent from the simulations, uncompetitive (against E-UTP-GlcNAc1P complex) inhibition of rxn-2 appears to cause maximal impact on overall GlmU rate under the physiologically relevant metabolite concentrations. As the next step, an *in vitro* assay must be designed to screen the compound libraries so as to identify uncompetitive (against E-UTP-GlcNAc1P complex) inhibitors targeting GlmU rxn-2. To select such inhibitors in an assay, significant fraction of the enzyme should exist in E-UTP-GlcNAc1P complex, which necessitates high concentration of UTP and GlcNAc1P in the assay mixture. The assay can be a decoupled assay, wherein each of the reactions of GlmU is assayed independent of the other GlmU reaction, or, coupled assay wherein both the GlmU reactions are assayed together and the readout is the synthesis of final end-product i.e. UDPGlcNAc or PPi. In a decoupled assay, deciding the initial concentrations of metabolites in the assay mixture is a trivial task because the metabolite concentrations can be kept low/high to select competitive/uncompetitive inhibitor respectively against a given GlmU reaction. However, in a coupled assay, the initial substrates that would be provided in the assay are: GlcN1P, AcCoA (i.e. substrates for rxn-1) and UTP (1^st^ substrate of rxn-2). The second substrate for rxn-2, GlcNAc1P, would be generated by the action of GlmU rxn-1 during the course of assay. Thus to maintain the concentration of GlcNAc1P in a desired range in the assay mixture, the initial concentrations of GlcN1P and AcCoA need to be chosen carefully such that GlcNAc1P concentration does not rise too high to cause significant product inhibition to rxn-1 and not drop too low that the formation of E-UTP-GlcNAc1P complex is hindered.

**Figure 6:**
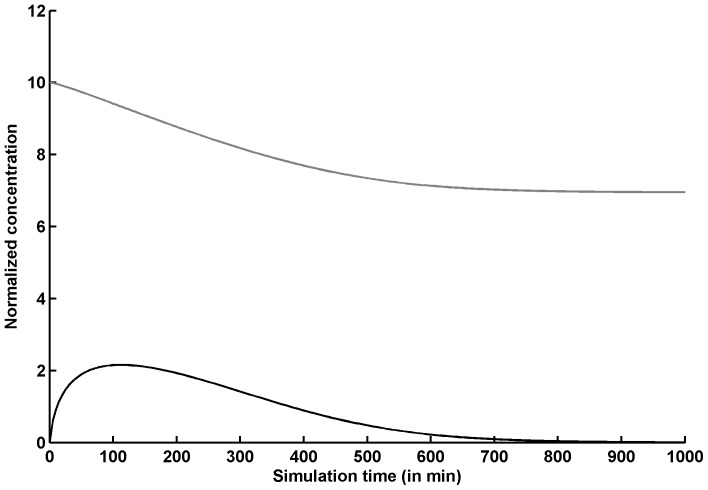
Dynamics of GlcNAc1P (black line) and UTP (gray line) normalised concentrations under the proposed assay condition. The normalized concentrations of both GlcNAc1P and UTP stay above 1 for significant portion of simulation time period, which is a favourable condition of assay for identifying uncompetitive (against E-UTP-GlcNAc1P complex) inhibitors against GlmU rxn-2.

The series of simulations indicate that it is best to maintain the initial concentrations of AcCoA and GlcN1P to twice of their *K_M_* values and of UTP to ten times of its *K_M_* value for the assay meant to screen for uncompetitive (against E-UTP-GlcNAc1P complex) inhibitors against rxn-2. With the said initial concentrations of AcCoA, GlcN1P and UTP, GlcNAc1P concentration hovers around *K_M_* to twice of *K_M_* and that of UTP hovers around 6 – 10 times of its *K_M_* value. Such higher than *K_M_* concentrations of GlcNAc1P and UTP would lead to rise in E-UTP-GlcNAc1P complex, which in turn, would favour the selection of uncompetitive (against E-UTP-GlcNAc1P complex) inhibitors against rxn-2 (see Figure 6). With higher initial concentrations of AcCoA and GlcN1P, the concentration of GlcNAc1P would rise. This will lead to high product inhibition on rxn-1, which is undesirable for the assay. It should be noted that the rate equations in this model are derived with rapid equilibrium assumption (see “Rate equations” section of “Methods” for details), which implies that the rate of catalysis is much slower than all other kinetic processes such as binding and dissociation of substrate, product and inhibitor. In case this assumption breaks down, the concentrations of GlmU, its substrates and the inhibitor would become very important in determining the *in vitro* potency of an inhibitor along with the factors such as binding and dissociation rate constants. However, whether the rapid equilibrium holds or not, the selection of uncompetitive (against E-UTP-GlcNAc1P complex) inhibitor would stipulate that the concentrations of UTP and GlNAc1P be high such that E-UTP-GlcNAc1P complex is available in the assay mixture to which the desired uncompetitive inhibitor would bind. The prediction of suitable assay condition would improve further once the details on *Mtu* associated kinetics of product inhibition become available.

### Conclusion

GlmU is an essential enzyme for the synthesis of an important precursor for peptidoglycan biosynthesis, hence is an attractive anti-TB drug target. In this study, kinetic modelling paradigm was used to simulate the dynamics of GlmU-catalyzed reactions and to predict the effect of inhibition of GlmU reactions on the overall GlmU rate. Based on the simulations, it was found that the inhibition of GlmU rxn-2, preferably with uncompetitive inhibitor against E-UTP-GlcNAc1P complex, would cause the maximal impact on GlmU rate under physiologically relevant metabolite concentrations. Further, the initial metabolite concentrations in a coupled biochemical assay mixture were also predicted so as to bias the assay towards the selection of this type of inhibitor. Thus the current work presents an example of the application of computational approaches in the early stages of drug discovery so as to make informed choices on the target and the preferred mode of inhibition such that the late-stage failures can be avoided or at least minimised. We premise that the present work can help identify high-affinity uncompetitive inhibitors of GlmU rxn-2 and further can potentiate their optimisation into lead drug molecules for the treatment of tuberculosis.

## Supporting Information

Table S1 **Derivation of GlmU kinetic parameters.**
(PDF)

Table S2 **Flux control coefficients of GlmU reactions under various metabolite concentrations.**
(PDF)
