# Supplementary material for: Kinetic Modelling of GlmU Reactions – Prioritization of Reaction for Therapeutic Application
Source: PLoS One. 2012 Aug 27;7(8):e43969. doi: 10.1371/journal.pone.0043969 (PMC3428340; doi:10.1371/journal.pone.0043969)
Supplement: Table S1 — Derivation of GlmU kinetic parameters. (PDF) [file pone.0043969.s001.pdf]

Table S1: Derivation of GlmU kinetic parameters

GlmU reaction	Kinetic parameter values: Experimentally-known or derived
Rxn-1	<u>*Vf* derivation</u><br>Zhou *et al.* [5] provide *Mtu* *Vf_rxn-1* and *Vf_rxn-2* values. *Vf_rxn-2* is known from AstraZeneca data (unpublished).<br>*Vf_rxn-1_literature* = 92.042µmol/min/mg protein [5]<br>*Vf_rxn-2_literature* = 3.382µmol/min/mg protein [5]<br>*Vf_rxn-2* = 0.0004mM/min (AstraZeneca data (unpublished) from *Mtu* (see computation in the next section))<br>Deriving a scaling factor from [5], *Vf_rxn-1* was computed as:<br>$$\text{Scaling factor, } x = \frac{Vf\_rxn-1\_literature}{Vf\_rxn-2\_literature}$$ $$Vf\_rxn\text{-}1 = x * Vf\_rxn\text{-}2$$<br>*Vf_rxn-1* = 0.01089mM/min<br><br>*Vr_rxn-1* = 0mM/min (Reverse reaction was not considered in the model)<br><u>K_M for substrates</u><br>K_M for substrates of rxn-1 were taken from [5]:<br>K_{GlcN1P_mtu} = 0.061mM [5]<br>K_{AcCoA_mtu} = 0.224mM [5]<br><br><u>K_M values for products</u><br>K_{CoA_mtu} = 10^9mM (assumed to be a large number)<br>$K_{GlcNAc1P\_mtu}$ = 0.003mM (derived from [22] and scaling factor described below)<br><br>To map the *Escherichia coli (Eco)* parameter value to *Mtu*, a scaling factor was derived based on K_M values of substrates of rxn-1 from *Mtu* and from *Eco*:<br>K_{GlcN1P_mtu} = 0.061mM [5]<br>K_{AcCoA_mtu} = 0.224mM [5]<br>K_{GlcN1P_eco} = 0.15mM [22]<br>K_{AcCoA_eco} = 0.6mM [22]<br>$$\text{Scaling factor, } x = Average\left[\left(\frac{K_{GlcN1P\_mtu}}{K_{GlcN1P\_eco}}\right) \& \left(\frac{K_{AcCoA\_mtu}}{K_{AcCoA\_eco}}\right)\right]$$ $$K_{GlcNAc1P\_mtu} = x * K_{GlcNAc1P\_eco}$$<br>Using $K_{GlcNAc1P\_eco}$ = 0.006977mM (derived from [22]), $K_{GlcNAc1P\_mtu}$ = 0.003mM
Rxn-2	<u>*Vf* derivation</u><br>*Vf_rxn-2* = 20pmol/min (AstraZeneca data (unpublished) from *Mtu*)<br>Reaction volume used for assay = 50µl<br>*Vf_rxn-2* (in concentration / time) = *Vf_rxn-2* (in amount / time) / Reaction volume<br>*Vf_rxn-2* = (20pmol/min) / (50µl)<br>*Vf_rxn-2* = 0.0004mM/min<br><br>*Vr_rxn-2* = 0mM/min (Reverse reaction was not considered in the model)<br><br><u>K_M values for substrates</u><br>K_{UTP_mtu} = 0.04mM (AstraZeneca data (unpublished) from *Mtu*)

$K_{GlcNAc1P\_mtu}$ = 0.04mM (AstraZeneca data (unpublished) from *Mtu*)
$K_{GlcNAc1P\_2\_mtu}$ = 0.04mM (taken equal to $K_{GlcNAc1P\_mtu}$)

Using parameter estimation functionality of COPASI [21], the parameter values were chosen such that they reproduce *in vitro* GlcNAc1P concentration response curve observed experimentally. The values used for simulations are provided below:
K_{UTP_mtu} = 0.04mM
$K_{GlcNAc1P\_mtu}$ = 0.033mM
$K_{GlcNAc1P\_2\_mtu}$ = 0.04mM (taken equal to $K_{GlcNAc1P\_mtu}$)

<u>K_M values for products</u>
K_{PPi_mtu} = 10^9mM (Assumed to be a large number)
$K_{UDPGlcNAc\_mtu}$ = 0.132mM (derived from [22] and scaling factor described below)
To map the *Eco* parameter value to *Mtu*, a scaling factor was derived based on K_M values of substrates of rxn-2 from *Mtu* and from *Eco*:
K_{UTP_mtu} = 0.04mM (AstraZeneca data (unpublished) from *Mtu*)
$K_{GlcNAc1P\_mtu}$ = 0.04mM (AstraZeneca data (unpublished) from *Mtu*)
K_{UTP_eco} = 0.1mM [22]
$K_{GlcNAc1P\_eco}$ = 0.07mM [22]

Scaling factor,

$$x = Average\left[\left(\frac{K_{UTP\_mtu}}{K_{UTP\_eco}}\right) \& \left(\frac{K_{GlcNAc1P\_mtu}}{K_{GlcNAc1P\_eco}}\right)\right]$$

$$K_{UDPGlcNAc\_mtu} = x * K_{UDPGlcNAc\_eco}$$

Using $K_{UDPGlcNAc\_eco}$ = 0.272727mM (derived from [22]), $K_{UDPGlcNAc\_mtu}$ = 0.132mM
