# Supplementary material for: Kinetic Modelling of GlmU Reactions – Prioritization of Reaction for Therapeutic Application
Source: PLoS One. 2012 Aug 27;7(8):e43969. doi: 10.1371/journal.pone.0043969 (PMC3428340; doi:10.1371/journal.pone.0043969)
Supplement: Table S2 — Flux control coefficients of GlmU reactions under various metabolite concentrations. (PDF) [file pone.0043969.s002.pdf]

Table S2: Flux control coefficients of GlmU reactions under various metabolite concentrations
Metabolite concentrations used for computation: Low (= $0.1xK_M$), Medium (= K_M), High (= $10xK_M$) and Intracellular levels; Coupled *in vivo* variant of the model used for computation

	**Control on overall GlmU reaction rate**			
Metabolite concentration	***Low***	***Medium***	***High***	***Intracellular***
Rxn-1 Flux Control Coefficient	0.5	0.3	0.0	0.0
Rxn-2 Flux Control Coefficient	0.5	0.7	1.0	1.0
